# Treating alcohol use disorder: recent advances in pharmacology and genomics

**DOI:** 10.1016/j.ebiom.2026.106258

**Published:** 2026-04-08

**Authors:** eBioMedicine

Alcohol use disorder (AUD) is a global public health challenge, with more than 110 million cases in 2021. It is a serious but preventable contributor to morbidity and mortality and is associated with substantial personal, societal, and economic burden. Even modest reductions in alcohol consumption can lead to meaningful health improvements, highlighting the importance of both prevention and supportive treatment strategies.

AUD is underdiagnosed and undertreated. Despite Food and Drug Administration (FDA)-approved medications being available in addition to non-pharmacological management, as little as 1.6% of patients with AUD in the USA are estimated to receive pharmacotherapy as part of their treatment. There are various contributing factors, including stigma, lack of public funding, and low efficacy of existing medications and contraindications such as hepatic or renal disease. Recent pharmacological and genomic research is offering new hope for the expansion of the available treatment options.

An exciting development in the AUD treatment landscape is glucagon-like peptide-1 (GLP-1) receptor agonists, a class of drugs originally developed for the treatment of type 2 diabetes and obesity. Increasing evidence has suggested that these drugs might also be beneficial in reducing alcohol consumption. Preclinical studies, published in *eBioMedicine* in July, 2023, May, 2025, and February, 2026 consistently showed that GLP-1R agonists reduced alcohol intake through modulation of reward pathways in the brain. Further preclinical work, published in *npj Metabolic Health and Disease* in September, 2025 found that GLP1-R agonists might also confer a more direct protective effect to the liver by reducing the production of the toxic metabolite acetaldehyde.

Studies in humans likewise support the use of GLP-1R agonists to reduce alcohol cravings and consumption. A nationwide registry study in Sweden, published in *JAMA Psychiatry**,* reported a decreased risk of alcohol-related hospitalisation; of note, this risk was lower for GLP-1R agonists than for approved AUD medications. In 2025, a small randomised clinical trial of patients receiving a low dose of GLP1-R agonist semaglutide reported a reduction in craving in the amount of alcohol consumed when compared with placebo. Larger, multi-centre trials are needed to continue to evaluate the repurposing of GLP-1R agonists for AUD.

The potential effect of GLP-1R agonists for AUD treatment is, however, tempered by access and affordability concerns, as has already been seen with their use in obesity management. This limitation raises the possibility of widening health inequalities, which is of particular concern given known treatment disparities associated with lower socioeconomic index and higher area deprivation index. Low-income and middle-income countries (LMICs) are expected to face an increasingly high burden of alcohol-related deaths in the coming years.

Although environmental and social factors represent major contributors to AUD risk, genetic factors also account for a substantial proportion of the risk, with heritability estimated to be around 50%. Genetic variation in alcohol metabolising genes (eg, *ADH1B, ADH1C*) has been associated with alcohol metabolism, alcohol consumption, and AUD. Polymorphisms in these genes also have broader implications for health beyond alcohol-related conditions. A phenome-wide association study, *published in eBioMedicine in 2024*, identified significant associations with known comorbid conditions, including cardiometabolic, immune, and gastrointestinal conditions. A September, 2025 study in *The American Journal of Psychiatry* confirmed that, although environmental factors such as education and annual household income explained most of the variance in AUD, polygenic risk was also a statistically significant predictor in both African and European ancestry groups.

Genomic studies have identified genes that might play a role in AUD, bringing not only an increased understanding of the underlying pathomechanism, but also the possibility to target new pathways for treatment development. A mendelian-randomisation study, published in *eBioMedicine* in October, 2025, identified 97 genes and 13 proteins with a probable casual role in AUD and strong enrichment for brain expression. A set of high-confidence genes with robust co-localisation evidence and replication across datasets were prioritised as potential AUD drug targets. The work represents a framework for genetically informed identification of potential targets, some of which might represent drug repurposing opportunities. However, functional work is necessary to validate and prioritise any new therapeutic targets.

Translating pharmacological and genomic research into accessible, effective interventions for AUD remains a major challenge, as meaningful progress will also require improved access to treatment, changes to public health policy, and increased health-care investment. *eBioMedicine* welcomes studies that contribute to the development of more effective strategies to prevent and treat AUD, ultimately reducing the substantial global health burden associated with alcohol consumption.

